# The Role of JAK-STAT-SOCS1 Axis in Tumorigenesis, Malignant Progression and Lymphatic Metastasis of Penile Cancer

**DOI:** 10.7150/ijms.95490

**Published:** 2024-04-29

**Authors:** Da-Ming Xu, Ling-Xiao Chen, Xiao-Yu Zhuang, Hui Han, Miao Mo

**Affiliations:** 1 State Key Laboratory of Oncology in South China, Guangdong Provincial Clinical Research Center for Cancer, Sun Yat-sen University Cancer Center, Guangzhou 510060, P. R. China.; 2 Department of Urology, Sun Yat-sen University Cancer Center, Guangzhou 510060, P.R. China.; 3 Department of Urology, Xiangya Hospital, Central South University, Changsha 410008, P.R. China.; 4 Department of Anesthesiology, Second Affiliated Hospital of Shantou University Medical College, Shantou 515041, P. R. China.

**Keywords:** JAK-STAT-SOCS1 Axis, Penile Cancer

## Abstract

**Background:** To uncover the potential significance of JAK-STAT-SOCS1 axis in penile cancer, our study was the pioneer in exploring the altered expression processes of JAK-STAT-SOCS1 axis in tumorigenesis, malignant progression and lymphatic metastasis of penile cancer.

**Methods:** In current study, the comprehensive analysis of JAK-STAT-SOCS1 axis in penile cancer was analyzed via multiple analysis approaches based on GSE196978 data, single-cell data (6 cancer samples) and bulk RNA data (7 cancer samples and 7 metastasis lymph nodes).

**Results:** Our study observed an altered molecular expression of JAK-STAT-SOCS1 axis during three different stages of penile cancer, from tumorigenesis to malignant progression to lymphatic metastasis. STAT4 was an important dominant molecule in penile cancer, which mediated the immunosuppressive tumor microenvironment by driving the apoptosis of cytotoxic T cell and was also a valuable biomarker of immune checkpoint inhibitor treatment response.

**Conclusions:** Our findings revealed that the complexity of JAK-STAT-SOCS1 axis and the predominant role of STAT4 in penile cancer, which can mediate tumorigenesis, malignant progression, and lymphatic metastasis. This insight provided valuable information for developing precise treatment strategies for patients with penile cancer.

## Introduction

Penile cancer is an exceedingly rare malignant urologic neoplasm with an overall cancer incidence of 0.5-0.94/100 000 males in the United States and Europe 1. Surgical treatment may be appropriate only for local disease, but patients with advanced penile cancer are often associated with inguinal or pelvic lymph node involvement. Limited by treatment options, survival outcomes among patients with advanced cancer substantially are very poor 2. In fact, patients with positive inguinal or pelvic lymph node metastasis have an overall survival of approximately 15% at 5 years 3. Established risk factors for penile cancer include phimosis, chronic inflammation in penis, lichen sclerosus, UV-A phototherapy, habit of smoking and poor socioeconomic status 4. Over 95% of penile cancers are diagnosed as penile squamous cell carcinoma (PSCC). The histological subtypes of PSCC include human papillomavirus (HPV)-independent PSCC (usual, pseudohyperplastic, pseudoglandular, verrucous, cuniculatum, papillary, sarcomatoid and mixed), HPV-associated PSCC (basaloid, warty, warty-basaloid, lymphoepitheliomalike and mixed) and other 1,5. In HPV-associated PSCC, high-risk HPV infection leads to uncontrolled cell proliferation by infecting penile epithelial cells and integrating viral genes (such as E6 and E7) 6,7. By contrast, in HPV-independent PSCC, chronic inflammation and TP53 mutation are the main causes of carcinogenesis of penile cancer, which can promote the progression of malignant phenotypes such as invasion and angiogenesis 8,9. Additionally, the oncogenic function of the PI3K-AKT-mTOR signaling pathway in penile cancer has been demonstrated 10. Notably, this rare malignancy makes a great therapeutic challenge. In penile cancer, understanding the underlying biological processes, cancer microenvironment and significant biomarkers that drive the cancer is the foundation for the development of effective treatment. Recently, more and more scattered evidence suggests the significance of JAK-STAT pathway in penile cancer 11,12. Simultaneously, JAK-STAT signaling axis has also been confirmed to be associated with cancer drug resistance 13. JAK-STAT pathway signal transduction is mainly mediated by extracellular and intracellular stepwise phosphorylation, which eventually binds to DNA regulatory elements and induces gene transcription 14. As mentioned, the research significance of JAK-STAT pathway in penile cancer has gradually emerged. Therefore, more sophisticated understanding of JAKs family, STATs family and their negative feedback regulators (SOCS1) will offer more opportunities for specificity therapy.

The novelty of our study lied in applying single-cell RNA sequencing experiment, bulk RNA sequencing experiment and other approaches to delineate the altered expression processes of JAK-STAT-SOCS1 axis in tumorigenesis, malignant progression and lymphatic metastasis of penile cancer. Collectively, our study was the first to make a comprehensive analysis of JAK-STAT-SOCS1 axis in penile cancer, thereby offering the new drug target for penile cancer treatment.

## Materials and Methods

### Differential expression analysis via Gene Expression Omnibus (GEO)

GSE196978 (6 tumor sample: GSM5906201, GSM5906202, GSM5906203, GSM5906204, GSM5906205, GSM5906206 and 6 normal penile tissue: GSM5906195, GSM5906196, GSM5906197, GSM5906198, GSM5906199, GSM5906200) were used to performed a comparation between penile cancer and normal penile tissue (p < 0.05) via R (4.2.2) and limma (3.54.0) packages.

### Single-cell RNA sequencing experiments

Patients: A total of 6 penile cancer patients, classified according to the cancer staging criteria of the 8th American Joint Committee on Cancer, were included in our study for Single-cell RNA-seq analysis, which were divided into two group: advanced cancer group (Stage IV malignancy, n = 4) and early cancer group (Stage I malignancy, n = 2).

Cell quality control: An equal volume of 0.4% trypan blue staining solution was added into a small amount of single cell suspension, and cell counting was performed utilizing Countess® II Automated Cell Counter. Finally, the viable cells concentration was need to adjust to an ideal concentration.

10X labeled cDNA fragments: Gel beads including the barcode information associate with cells and enzymes, get into the reservoir and are allowed to be separated by oil to make a formation of gel-beads-in-emulsions (GEMs). Subsequently, gel bead lysis released capture sequences with barcode information. The cDNA fragments were reverse transcribed, and samples were labeled. PCR amplification was allowed to perform based on cDNA template. Combining all the GEMs products to construct a standard sequencing library.

Library construction of standard sequencing library: The cDNA fragments were cut into 200 to 300 bp fragments through enzyme digestion. The routine steps of next-generation sequencing library construction were performed. PCR was used to amplify the DNA library

Sequencing of libraries: The constructed library was sequenced using PE150 mode of Illumina sequencing platform.

### Bulk RNA sequencing experiments

Patients: A total of 7 penile cancer patients with lymphatic metastasis were included in our study for Bulk RNA-seq analysis, which were divided into two group: Primary tumor site group (Penile cancer, n = 7) and their paired lymphatic metastasis group (Metastatic lymph node, n = 7).

RNA Sample Preparation: After total RNA extraction from the sample, the mRNA was enriched by removing rRNA using a conventional kit. The enriched mRNA was reverse transcribed to form double-stranded cDNA. After repairing the double ends of cDNA, the adaptor was added and the library was constructed by PCR amplification.

Library preparation: Eukaryotic mRNA with polyA tails was enriched using magnetic beads with Oligo and disrupted by ultrasound. In the M-MuLV reverse transcriptase system, cDNA double-strands are synthesized, followed by end repair, A-tail addition, and connection of an A sequencing adapter. Approximately 200bp cDNA fragments were screened using AMPure XP beads, and PCR products were allowed to purify again using AMPure XP beads to obtain the final library.

### Molecular interaction analysis

InAct (https://www.ebi.ac.uk/intact/home) is a database for molecular interaction tool based on previous literature curation. We made a molecular interaction analysis of STAT4 via InAct to reveal its important accessory molecules. Then. STAT4 and its 26 accessory protein molecules in homo sapiens were selected to perform an immune infiltration analysis through GSEA platform 15.

### Prediction of immune checkpoint inhibitor treatment response

ROC Plotter is a database which has the ability to evaluate the efficacy of immunotherapy based on gene expression 16. We conducted an exploratory investigation to gather the relationship data between STAT4 expression and the therapy efficacy of anti PD-1 therapy, containing pembrolizumab and nivolumab regimens, anti PD-L1 therapy, such as atezolizumab regimen and anti CTLA4 therapy, like ipilimumab regimen, via ROC Plotter.

### Single cell trajectories analysis

Single cell trajectory in T cell subpopulation was measured by Monocle tool (version2.10.1) to reveal the expression abundance and expression status of target genes based on pseudotime variation 17,18.

### Function enrichment analysis

Gene ontology (GO) is an international authoritative database for the analysis of gene function which has three different analysis levels to reveal the gene properties 19. KEGG is a pathways database for the analysis of gene signaling pathways 20. Our study made a GO and KEGG enrichment analysis of upregulated genes in cytotoxic T cell which was the T cell subgroup with the strongest expression of STAT4.

## Results

### Differential expression between penile cancer and normal penile tissue

**Figure 1:**
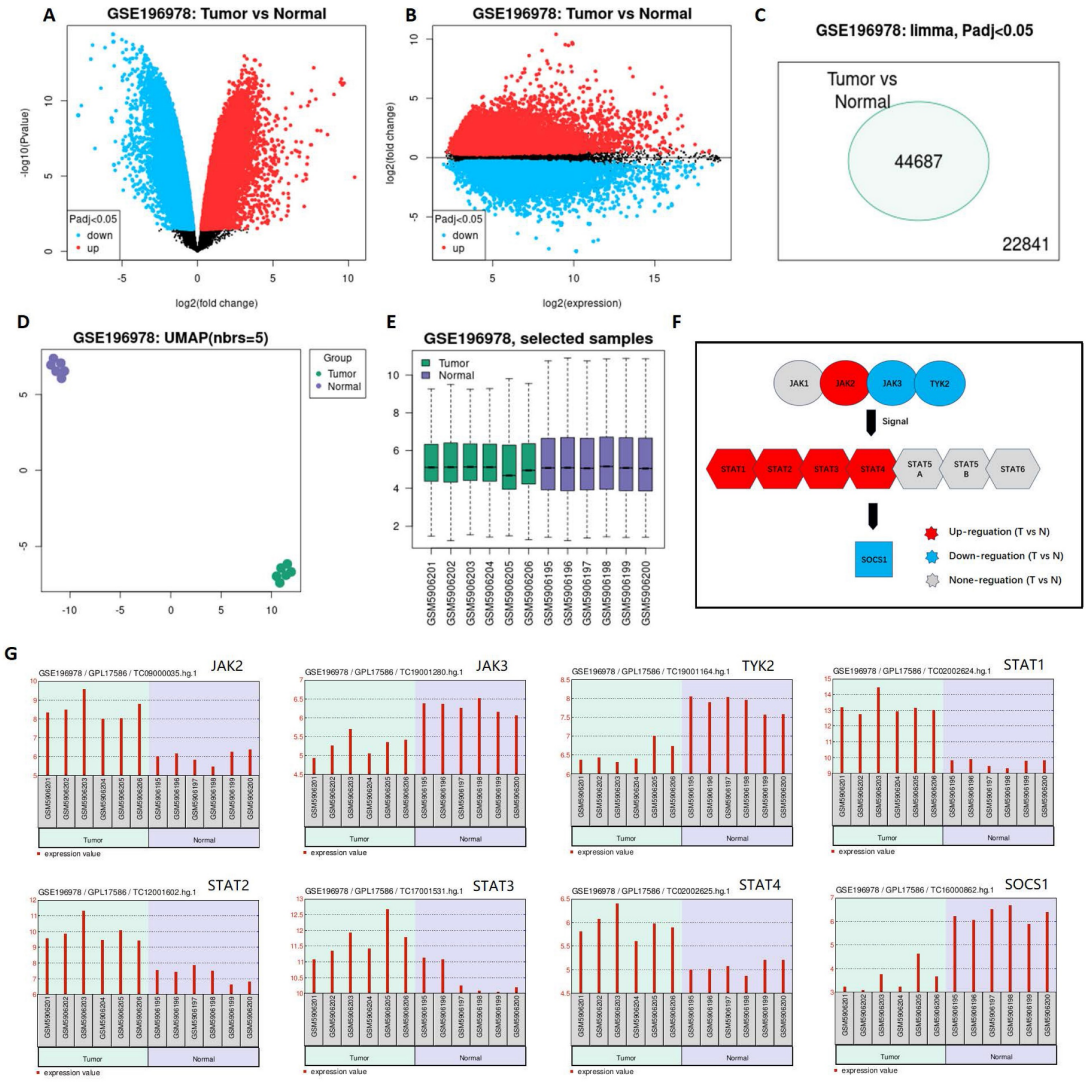
**Differential expression between penile cancer and normal penile tissue based on GSE196978 data.** (A and B: Differences in gene expression via volcano plot and mean-difference plot; C: Venn diagram for 44687 significantly different genes; D and E: UMAP plot and box plot of GSE196978; F The diagram of JAK-STAT-SOCS1 axis expression; G: The up-regulation expression of JAK2, STAT1, STAT2, STAT3, STAT4 and the down-expression of JAK3 TYK2, SOCS1 in penile cancer comparing with normal penile tissue.)

The result demonstrated that the differential expression between penile cancer and normal penile tissue **(Figure 1)**. Figure 1A and 1B indicated the differences in gene expression via volcano plot and mean-difference plot. Figure 1C draw a Venn diagram to compare two samples, showing 44687 significantly different genes (p <0.05). Figure 1D and 1E indicated 12 samples' UMAP plot and box plot. Figure 1F displayed that the up-regulation expression of JAK2, STAT1, STAT2, STAT3, STAT4 and the down-expression of JAK3 TYK2, SOCS1 in penile cancer comparing with normal penile tissue, which had a statistical significance. Figure 1G revealed the detail gene expression of JAK2, STAT1, STAT2, STAT3, STAT4, JAK3 TYK2 and SOCS1 in each sample.

### Single-cell RNA sequencing was used to reveal the difference between advanced penile cancer and early penile cancer

**Figure 2:**
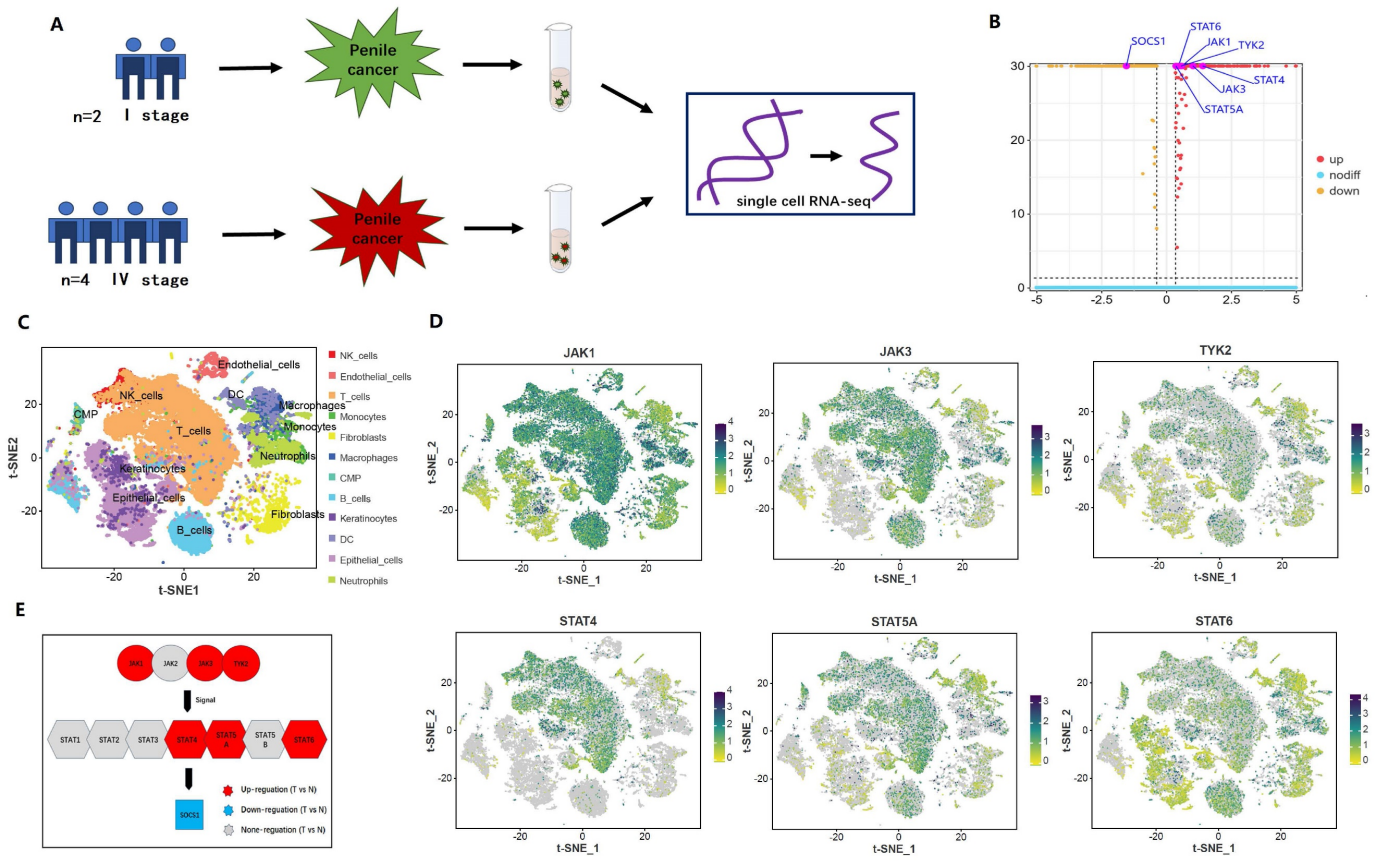
** Difference between advanced (IV stage) and early (I stage) penile cancer based on single-cell RNA sequencing.** (A: The experimental procedure of single-cell RNA sequencing; B: The up-regulation expression of JAK1, JAK3, TYK2, STAT4, STAT5A, STAT6 and the down-expression of SOCS1 in advanced penile cancer comparing with early penile cancer; C: Tumor microenvironment landscape of six penile cancer specimens; D: The expression degree of JAK1, JAK3, TYK2, STAT4, STAT5A and STAT6 in tumor microenvironment; E: The diagram of JAK-STAT-SOCS1 axis expression.)

The result indicated that the differential expression between advanced penile cancer (IV stage) and early penile cancer (I stage) **(Figure 2)**. Figure 2A showed the experimental procedure of Single-cell RNA sequencing. Figure 2B displayed that the upregulated expression of JAK1, JAK3, TYK2, STAT4, STAT5A, STAT6 and the down-expression of SOCS1 in advanced penile cancer comparing with early penile cancer, which had a statistical significance. Figure 2C mapped the tumor microenvironment of six penile cancer specimens, mainly including T cells, B cells, NK cells, Endothelial cells, DC, Macrophages, Monocytes, Neutrophils, Fibroblasts, Keratinocytes, Epithelial cells and CMP. Figure 2D indicated that the expression degree of JAK1, JAK3, TYK2, STAT4, STAT5A and STAT6 in tumor microenvironment. Figure 2E displayed the differences in JAK-STAT-SOCS1 Axis expression between early and advanced penile cancer through a schematic diagram.

### Bulk RNA sequencing was used to reveal the difference between penile cancer and their paired metastatic lymph nodes

**Figure 3:**
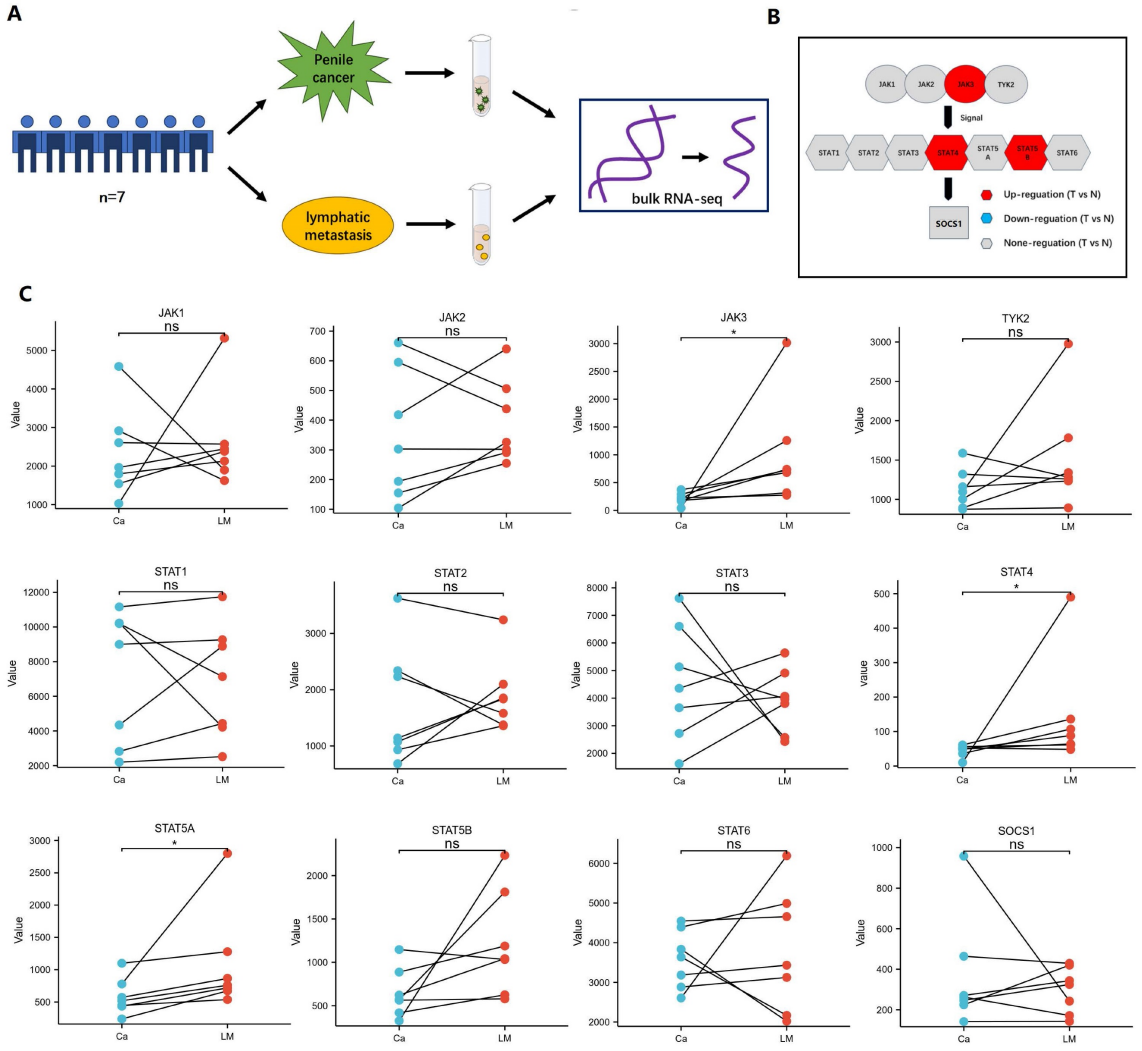
** Difference between penile cancer and the paired metastatic lymph nodes based on bulk RNA sequencing.** (A: The experimental procedure of Bulk RNA sequencing; B: The diagram of JAK-STAT-SOCS1 axis expression; C: The up-expression of JAK3, STAT4 and STAT5A in metastatic lymph nodes comparing with primary penile cancer.)

The result documented that the differential expression between penile cancer and the paired metastatic lymph nodes **(Figure 3)**. Figure 3A showed the experimental procedure of bulk RNA sequencing. Figure 3B and 3C displayed that the different expression level between penile cancer and their metastatic lymph nodes. There was a noticeable difference in the expression of JAK3, STAT4 and STAT5A between primary penile cancer and their metastatic lymph nodes.

### Comprehensive analysis of STAT4 and its 26 accessory protein molecules in immune infiltration and immune checkpoint inhibitor response

**Figure 4:**
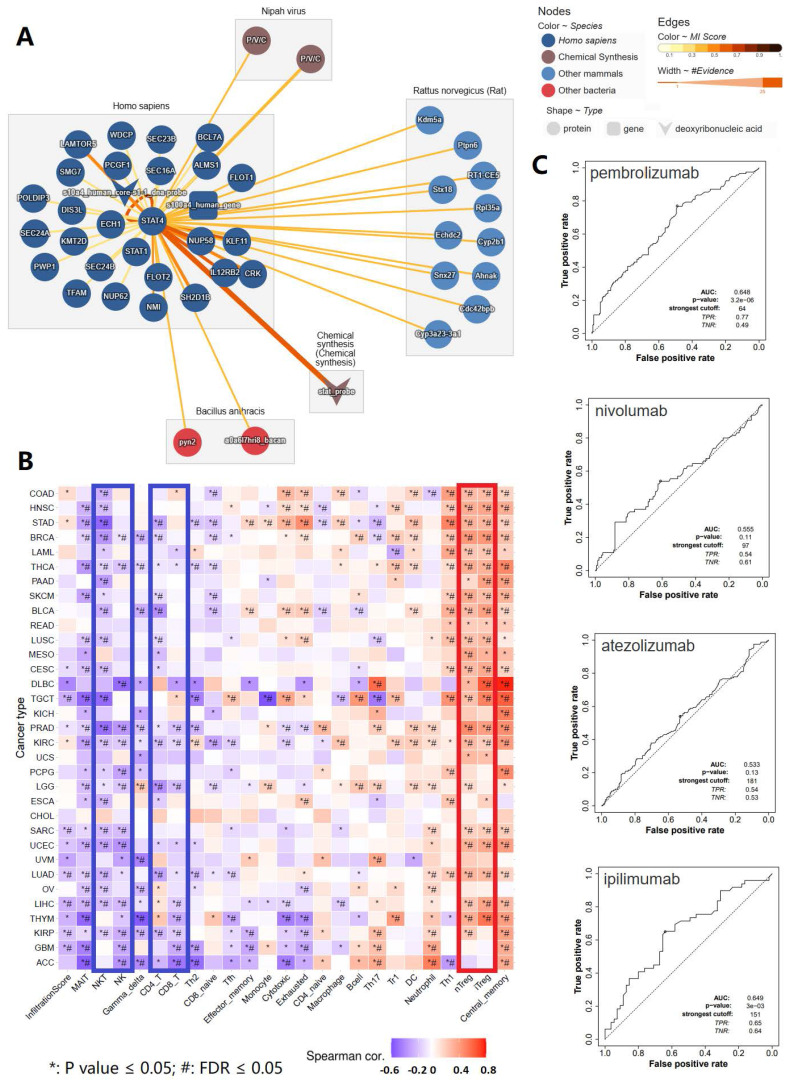
** Comprehensive analysis of STAT4 and its 26 accessory protein molecules in immune infiltration and immune checkpoint inhibitor response.** (A: The molecular interaction network of STAT4; B: The immune infiltration analysis of STAT4 and its 26 accessory protein molecules [POLDIP3, SEC24A, PWP1, TFAM, LAMTOR5, SMG7, DIS3L, KMT2D, SEC24B, NUP62, NMI, WDCP, PCGF1, ECH1, STAT1, FLOT2, SH2D1B, SEC23B, SEC16A, NUP58, IL12RB2, BCL7A, ALMS1, FLOT1, KLF11, CRK] in homo sapiens; C: The association between STAT4 expression and immune checkpoint inhibitors response.)

As we discovered in our current study, STAT4 appeared to be a protagonist in mediating tumorigenesis, malignant progression and lymphatic metastasis of penile cancer. Therefore, our subsequent research focused on STAT4. In order to make a comprehensive investigation to reveal the mechanism of STAT4 in penile cancer, molecular interaction analysis of STAT4 were applied to reveal its important accessory molecules in our study** (Figure 4)**. Figure 4A showed the molecular interaction network of STAT4. Figure 4B displayed that the immune infiltration analysis of STAT4 and its 26 accessory protein molecules (POLDIP3, SEC24A, PWP1, TFAM, LAMTOR5, SMG7, DIS3L, KMT2D, SEC24B, NUP62, NMI, WDCP, PCGF1, ECH1, STAT1, FLOT2, SH2D1B, SEC23B, SEC16A, NUP58, IL12RB2, BCL7A, ALMS1, FLOT1, KLF11, CRK) in homo sapiens, which were contributed to wake up the suppressor T cells and silence antitumor cells in pan cancers, thereby forming an immunosuppressive tumor microenvironment. Figure 4C found that STAT4 was associated with the response to anti PD-1 therapy (pembrolizumab) and anti CTLA4 therapy (ipilimumab) respectively.

### Comprehensive analysis of T cell subpopulation in tumor microenvironment

**Figure 5:**
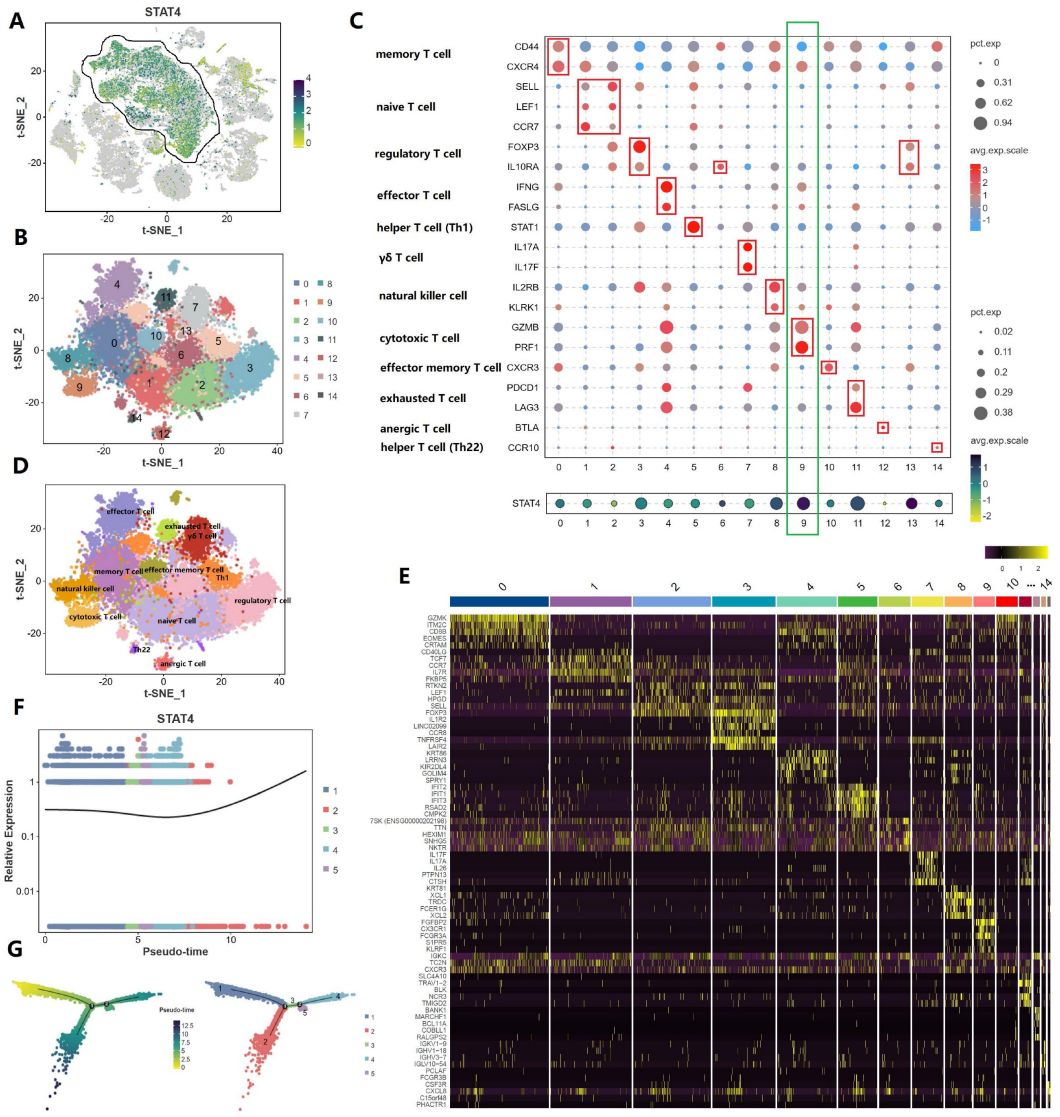
** Comprehensive analysis of T cell subpopulation in tumor microenvironment.** (A and B: Regrouping of T cell subpopulation; C and D: Annotation of T cell subpopulation by marker genes; E: The top 5 upregulated genes in each cluster; F: The single cell trajectory of STAT4; G: The expression abundance and expression status of T cell subpopulation based on pseudo-time variation.)

From the previously mentioned results, STAT4 was mainly expressed in T cell subpopulation in the immune microenvironment. Therefore, we make a comprehensive analysis of T cell subpopulation **(Figure 5)**. Figure 5A and 5B regrouped T cell subpopulation into 14 clusters. Figure 5C and 5D made the annotation of T cell subpopulation by marker genes (memory T cell: CD44,CXCR4; naïve T cell: SELL, LEF1, CCR7; regulatory T cell: FOXP3, IL10RA; effector T cell: IFNG, FASLG; helper T cell [Th1]: STAT1; yδ T cell:IL17A, IL17F; natural killer cell: IL2RB, KLRK1; cytotoxic T cell: GZMB, PRF1; effector memory T cell: CXCR3; exhausted T cell: PDCD1, LAG3; anergic T cell: BTLA; helper T cell [Th22]:CCR10). Notedly, STAT4 expression was highest in cluster 9, suggesting that it was associated with cytotoxic T cell. Figure 5E demonstrated that the top 5 upregulated genes in each cluster. Figure 5F applied the single cell trajectory analysis for STAT4, which was suggested that STAT4 expression in penile cancer gradually increased with time. Figure 5G revealed the expression abundance and expression status of T cell subpopulation based on pseudo-time variation.

### Function enrichment analysis of cluster 9 (cytotoxic T cell)

**Figure 6:**
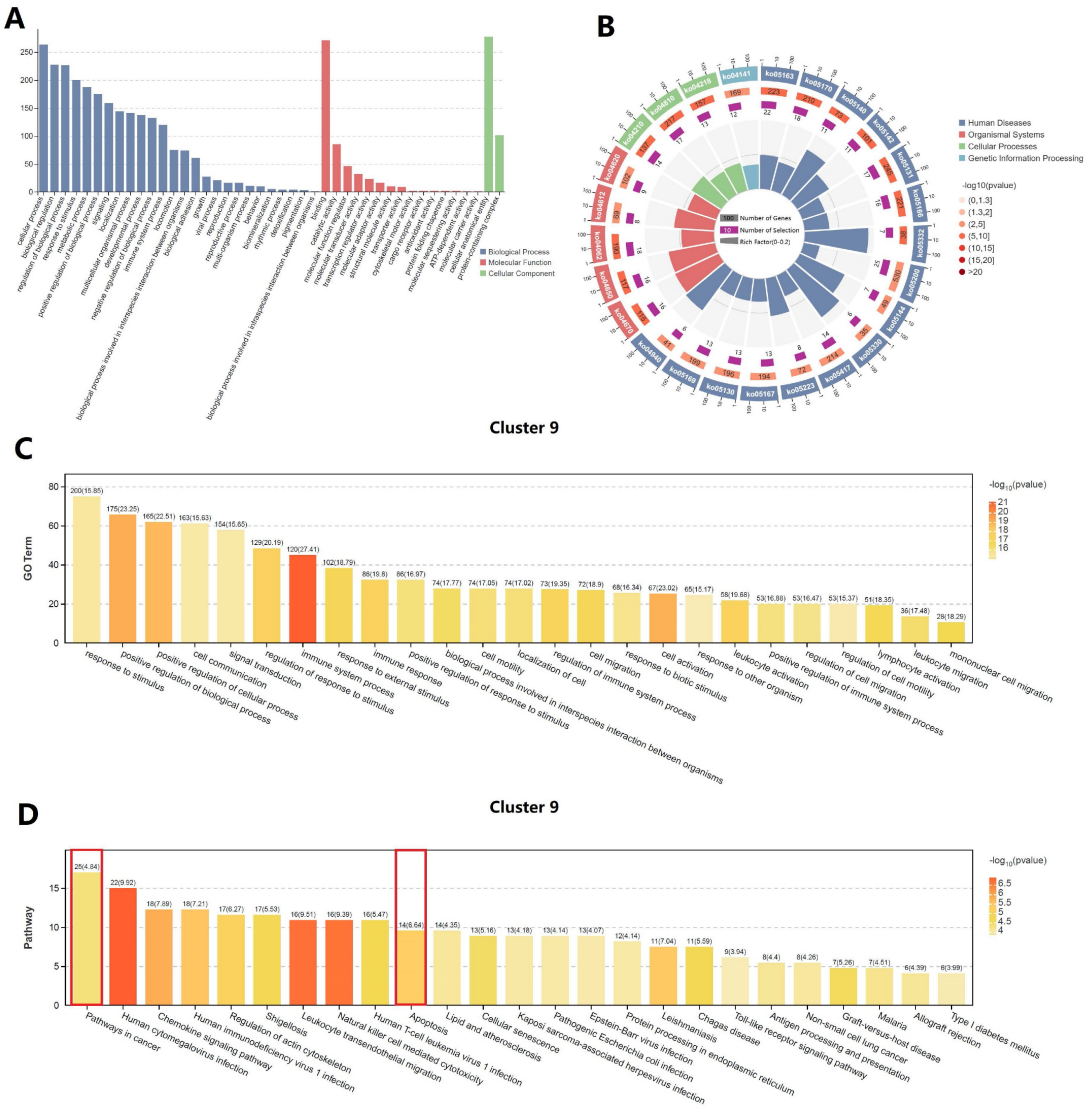
** Function enrichment analysis of cluster 9 (cytotoxic T cell).** (A: GO classification bar chart; B: KEGG enrichment circle diagram; C: GO enrichment analysis of cluster 9; D: KEGG enrichment analysis of cluster 9.)

GO and KEGG enrichment analysis of the upregulated genes in cluster 9 was indicated two important terms, “pathway in cancer” and “apoptosis” **(Figure 6)**. Therefore, we inferred that cytotoxic T cells in penile cancer were in an active state of apoptosis and their ability to destroy cancer cells was diminished. At the same time, the previous results of our study suggested the existence of regulatory T cell and exhausted T cell, which further proved that the immunosuppressive tumor microenvironment in penile cancer, thereby promoting the malignant progression and escape of cancer cells.

## Discussion

Due to the rare characteristics of penile cancer, the progression landscape of malignancy remains largely unknown, which limits our approach to accurately evaluate the progression, prognosis, and treatment options of this disease. Penile cancer, as a malignant tumor that seriously affects men's physical and mental health, family harmony and social stability, its prognosis is closely related to clinical and histological characteristics 21. The most essential prognostic factor affecting patient survival is the status of lymph node metastasis, with the 5-year cancer-specific survival rate for patients with N0 to N3 dropped from 95% to 35% 22. In addition, the prognosis of distant metastasis in penile cancer is even worse 23. Thus, in order to have a better understanding, the potential mechanisms of occurrence, progression and tumor metastasis of penile cancer were urgently needed to be investigated and explored. Currently, research on biomarkers helped to gain a deeper understanding of individuals with penile cancer who are at high risk of metastatic progression 24. At the same time, designing targeted therapy strategies aimed at inhibiting metastatic biomarkers could hold promise for improving patient prognosis. Our study provided important supplementary contributions to this field.

The JAK-STAT signaling was first discovered in 1957, and after approximately 30 years of exploration, there have been landmark discoveries in the field of cancer 14. The putative biological process is that cytokines bind to their receptors and can activate the JAKs with associated-receptor. Subsequently these JAKs can activate STATs by phosphorylating. The dimers, formed by tyrosine-phosphorylated STATs, are allowed to translocate to the nucleus and further combine with target DNA sequences, thereby regulating gene expression 25,26. In term of tumorigenesis, Huang et al. revealed that the activation of JAK-STAT axis can promote bladder cancer formation, and JAK-STAT inhibitors can significantly inhibit tumor activation 27. In term of metastases, Wang et al. found that JAK2 and STAT3 can contribute to cancer cell proliferation and migration in colorectal cancer 28. In term of treatment, JAK-STAT inflammatory signaling was enhanced in prostate cancer tumor cells which can lead to drug resistance 13, 29. JAK-STAT axis is a core signaling axis in a wide variety of t cancer 30. A previous pan cancer study showed that although all STATs follow similar molecular activation pathways, different STATs differed in their involvement in carcinogenesis 31. In current study, we observed an altered expression of JAK-STAT-SOCS1 axis during three different stages of penile cancer, from tumorigenesis to malignant progression to lymphatic metastasis. Notably, STAT4 was an important dominant player in the three-phase process. Therefore, it was reasonable to infer that the STAT4 was an essential biomarker of penile cancer.

During tumorigenesis phase of penile cancer, the expression evolution of JAK-STAT-SOCS1 axis was characterized by the upregulation of JAK2, STAT1, STAT2, STAT3 and STAT4. Previous evidence suggested that the combined activation of the JAK2-STAT3 signal was frequently found in various solid cancer. Persistently activated JAK2-STAT3 signal promotes cancer cells proliferation and survival, and also induces angiogenesis and immunosuppression in the tumor microenvironment 31,32. The deficiency of STAT2 was proved to inhibit tumorigenesis due to low levels of inflammatory factors 33. During malignant progression phase of penile cancer, the expression evolution of JAK-STAT-SOCS1 axis was characterized by the upregulation of JAK1, JAK3, STAT4, STAT5A and STAT6. Malignant signaling JAK1 can catalyze glycosylation and maintain stability of PD-L1 to suppress the antitumor effect of immune microenvironment 34. Yuan et al. found that JAK3 had an ability to promote the cancer malignant progression by cooperating to T cells 35. By contrast, STAT6 was documented to drive the M2 macrophage polarization to attenuate antitumor response 36. Additionally, the overexpression of STAT5A was significant related to cancer proliferation and invasion 37. During lymph node metastasis phase of penile cancer, the expression evolution of JAK-STAT-SOCS1 axis was characterized by the upregulation of JAK3, STAT4 and STAT5B. We noted that JAK3 has been reported to participate in cancer metastasis through phospholipase D 38. Similarly, STAT5B has been found to have an ability to promote tumor growth and metastasis 39. These findings firstly provide valuable implications to halt the progress of penile cancer by targeting the JAK-STAT-SOCS1 axis.

A growing number of studies have identified that STAT4 was closely related to the poor prognosis of various solid cancers 40. Study have found a robust correlation between immune checkpoint and STAT pathways, especially STAT4, in glioblastomas 41. Other scholars have also demonstrated that STAT4 can increased the expression of PD-1, enhancing the anti-cancer efficacy of immune checkpoint inhibitor therapy in prostate cancer 42. STAT4 and its 26 partner genes were mainly related to the activation of suppressor T cells and the inhibition of anti-tumor immune cells, such as CD4+ T cell, CD8+ T cell, NK and NKT, thereby forming an immunosuppressive tumor microenvironment, leading to cancer occurrence, progression and metastasis. This is consistent with what we observed in tumor microenvironment of penile cancer. We speculated that the overexpression of STAT4 contributed to make the formation of an immunosuppressive microenvironment in penile cancer and further promote the malignant progression. Importantly, we also found that STAT4 was associated with the response to two immune checkpoint inhibitors, including anti PD-1 therapy (pembrolizumab) and anti CTLA4 therapy (ipilimumab). We further hypothesized that STAT4 overexpression may enhance the expression of PD-1 or CTLA4, which suggested its potential as an important predictive biomarker for immunotherapy response in penile cancer patients.

## Conclusion

In summary, our findings revealed that the complexity of JAK-STAT-SOCS1 axis and the predominant role of STAT4 in penile cancer, which can mediate tumorigenesis, malignant progression, and lymphatic metastasis. This insight provided valuable information for developing precise treatment strategies for patients with penile cancer.
